# Pathogenesis and outcome of VA1 astrovirus infection in the human brain are defined by disruption of neural functions and imbalanced host immune responses

**DOI:** 10.1371/journal.ppat.1011544

**Published:** 2023-08-18

**Authors:** Olga A. Maximova, Melodie L. Weller, Tammy Krogmann, Daniel E. Sturdevant, Stacy Ricklefs, Kimmo Virtaneva, Craig Martens, Kurt Wollenberg, Mahnaz Minai, Ian N. Moore, Craig S. Sauter, Juliet N. Barker, W. Ian Lipkin, Danielle Seilhean, Avindra Nath, Jeffrey I. Cohen

**Affiliations:** 1 Laboratory of Infectious Diseases, National Institute of Allergy and Infectious Diseases, National Institutes of Health, Bethesda, Maryland, United States of America; 2 Secretory Physiology Section, Molecular Physiology and Therapeutics Branch, National Institute of Dental and Craniofacial Research, National Institutes of Health, Bethesda, Maryland, United States of America; 3 Research Technologies Branch, Genomics Unit, National Institute of Allergy and Infectious Diseases, National Institutes of Health, Hamilton, Montana, United States of America; 4 Bioinformatics and Computational Biosciences Branch, Office of Cyber Infrastructure and Computational Biology, National Institute of Allergy and Infectious Diseases, National Institutes of Health, Bethesda, Maryland, United States of America; 5 Infectious Disease Pathogenesis Section, Comparative Medicine Branch, National Institute of Allergy and Infectious Diseases, National Institutes of Health, Bethesda, Maryland, United States of America; 6 Department of Hematology and Medical Oncology, Taussig Cancer Institute, Cleveland Clinic, Cleveland, Ohio, United States of America; 7 Adult Bone Marrow Transplant Service, Department of Medicine, Memorial Sloan Kettering Cancer Center, New York, New York, United States of America; 8 Center for Infection and Immunity, Mailman School of Public Health, Columbia University, New York, New York, United States of America; 9 Sorbonne Université, Paris, France; 10 Infections of the Nervous System Section, National Institute of Neurological Disorders and Stroke, National Institutes of Health, Bethesda, Maryland, United States of America; University of Pennsylvania School of Medicine, UNITED STATES

## Abstract

Astroviruses (AstVs) can cause of severe infection of the central nervous system (CNS) in immunocompromised individuals. Here, we identified a human AstV of the VA1 genotype, HAstV-NIH, as the cause of fatal encephalitis in an immunocompromised adult. We investigated the cells targeted by AstV, neurophysiological changes, and host responses by analyzing gene expression, protein expression, and cellular morphology in brain tissue from three cases of AstV neurologic disease (AstV-ND). We demonstrate that neurons are the principal cells targeted by AstV in the brain and that the cerebellum and brainstem have the highest burden of infection. Detection of VA1 AstV in interconnected brain structures such as thalamus, deep cerebellar nuclei, Purkinje cells, and pontine nuclei indicates that AstV may spread between connected neurons transsynaptically. We found transcriptional dysregulation of neural functions and disruption of both excitatory and inhibitory synaptic innervation of infected neurons. Importantly, transcriptional dysregulation of neural functions occurred in fatal cases, but not in a patient that survived AstV-ND. We show that the innate, but not adaptive immune response was transcriptionally driving host defense in the brain of immunocompromised patients with AstV-ND. Both transcriptome and molecular pathology studies showed that most of the cellular changes were associated with CNS-intrinsic cells involved in phagocytosis and injury repair (microglia, perivascular/parenchymal border macrophages, and astrocytes), but not CNS-extrinsic cells (T and B cells), suggesting an imbalance of innate and adaptive immune responses to AstV infection in the brain as a result of the underlying immunodeficiencies. These results show that VA1 AstV infection of the brain in immunocompromised humans is associated with imbalanced host defense responses, disruption of neuronal somatodendritic compartments and synapses and increased phagocytic cellular activity. Improved understanding of the response to viral infections of the human CNS may provide clues for how to manipulate these processes to improve outcomes.

## Author summary

Immunocompromised individuals are at increased risk of severe viral infection of the central nervous system (CNS) due to a failure to contain infection before it reaches the brain. Here, we identified the VA1-clade astrovirus infection as the cause of fatal encephalitis in an immunocompromised patient and show that it targeted neurons in the brain. Study of brain tissues from three cases of astrovirus encephalitis in immunocompromised patients showed that the disease was associated with impairment of many functions of neurons and disruption of both excitatory and inhibitory synapses that form neuronal connections. Further analyses showed that the early innate, but not later adaptive immune response was driving antiviral host defense response in the brain. Activation of the CNS-resident cells important for phagocytosis, homeostasis, and repair (microglia, macrophages, and astrocytes) was evident, with little activity of the adaptive peripheral immune cells (T cells and B cells). These results demonstrate that the pathogenesis and outcome of astrovirus infection in the brain of patients with underlying immunodeficiencies is defined by an imbalance of different arms of the antiviral host defense system and disruption of functions of infected neurons. These findings suggest pathways that can be targeted to improve outcome of viral CNS infections.

## Introduction

Infections of the central nervous system (CNS) in humans cause significant morbidity and mortality and are increasing due to spread of pathogens to new locations, emergence of new pathogens, and an increasing number of patients with immunodeficiencies. Primary (genetic) and secondary (acquired) immunodeficiencies [1] are associated with an increased susceptibility to CNS infections [2–4]. Although the mammalian CNS is protected from most virus infections by multiple barriers [5–7], numerous viruses can enter the CNS when infection is poorly controlled in the peripheral tissues due to impaired host immunity [4]. Viruses that escape immune control at the initial sites of infection can spread to the CNS, where they can cause devastating neurological disease (ND) due to virus replication in cells (e.g., neurons) [5,8], overreacting immune responses [2,5], or disruption of the immune-neural-synaptic axis [9]. Astroviruses (AstVs) are increasingly recognized as a cause of CNS infections in both humans and animals, often causing fatal meningoencephalitis [10–12]. AstVs are well-established causes of viral gastroenteritis and their recovery from feces of asymptomatic children and mammals suggest that they may be a part of gut virome [12].

To date, twelve publications describe 14 human cases of AstV neurological disease (AstV-ND) [13–24] with the neuropathological findings reported only for 7 cases (postmortem findings from 5 cases [13,14,16,17,19] and biopsy findings from 2 cases [15,22]). We reviewed these publications and summarized the reported underlying premorbid conditions, types of infected cells, and pathology within the CNS (S1 Table). This review showed that AstV-ND predominantly occurs in humans with primary immunodeficiencies or hematologic malignancies undergoing immunosuppressive treatments. Whether CNS AstV infections in animals are associated with any underlying disease or immunodeficiencies is unknown [25–33]. Neurons, in both humans and animals, are the most often reported cells harboring either AstV RNA, AstV capsid protein, and/or viral paracrystalline arrays [16,17,25–30] (S1 Table). One report detected AstV capsid protein in one neuron from a biopsy specimen and in cells thought to be hypertrophic astrocytes in the postmortem brain tissue of an immunocompromised boy who died from AstV-ND [13]. Pathological changes associated with CNS AstV infection appear to be nonspecific and typical of many other viral CNS infections [8,34]. These encompass responses of resident CNS cells that manifest as astrocytosis, microgliosis, and neuronal degeneration/loss. The adaptive immune responses in the brain of immunocompromised patients with AstV-ND are most likely impaired (S1 Table). AstV infection of the CNS was fatal in most immunocompromised patients and was predominantly associated with the human astroviruses of the VA1 lineage (S1 Table). In contrast, patients without reported underlying immunodeficiencies have recovered from AstV-ND [20,21]. How impairment of the adaptive immunity contributes to the pathogenesis of AstV-ND and influences the disease outcome requires further study.

Here, we identified HAstV-NIH of the VA1 genotype as the cause of fatal encephalitis in an immunocompromised adult. We analyzed brain tissue from this patient and two other immunocompromised patients with AstV-ND using an integrative approach to study neurophysiological changes and host defense responses to infection at the level of transcriptional regulation, protein expression, and cell morphology/topology/function.

## Results

### Astrovirus HAstV-NIH infects neurons in directly connected structures of the human brain, indicating transsynaptic spread

We investigated the etiology of encephalitis in a 58-year-old-man who had undergone umbilical cord blood transplant for lymphoma and received immunosuppression for graft-versus-host disease (see Materials and Methods for the case report). RNA was isolated from a frozen portion of the patient’s brain and hybridized to a virus chip containing over 3000 probes for viral families and the predominant virus sequences detected (> 10,000-fold change over normal control) belonged to the family of *Astroviridae* (S1A Fig). We confirmed this finding by detecting AstV sequences by PCR in another portion of the patient’s brain tissue, but not in a normal control human brain (S1B Fig), and by finding AstV RNA using *in situ* hybridization in the brain tissue from the patient, but not from a normal control brain (S1C and S1D Fig). The virus was designated as HAstV-NIH (Genbank accession number OP293097.2).

Sequencing of the capsid precursor protein gene of HAstV-NIH, followed by phylogenetic analysis (S2 Fig), placed this virus within the VA1-HMO (Virginia/Human-Mink-Ovine-like) clade (reviewed in [12]), together with four other neuropathogenic AstVs: HAstV-PS [13], HAstV-VA1/HMO-C-PA [15], HAstV-SG [16], and HAstV-VA1/HMO-C-UK1(a) [17]. VA1/HMO-C AstVs, unlike classic astroviruses HAstV 1–8, are neuropathogenic, particularly in immunocompromised patients (reviewed in [12]).

**Fig 1:**
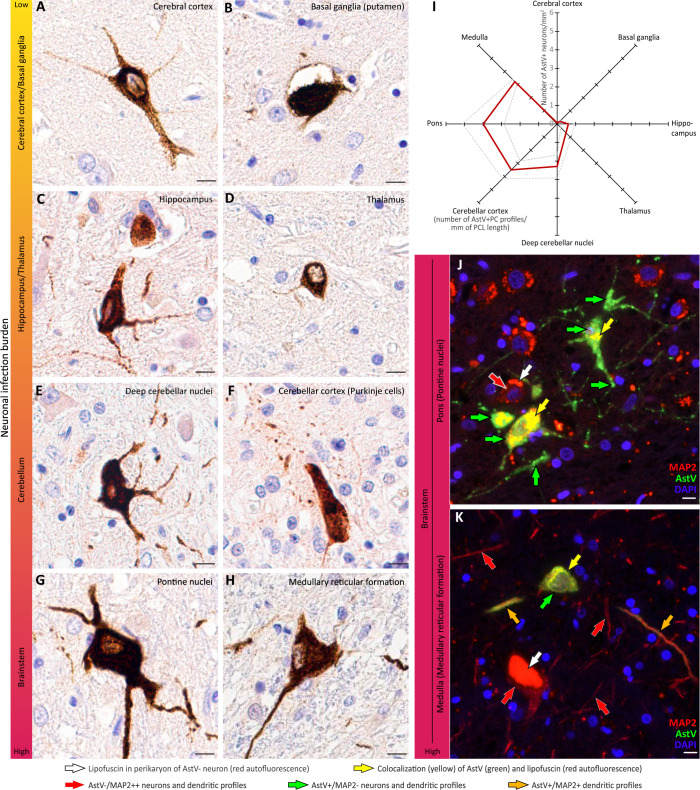
HAstV-NIH infects neurons with the highest burden in the cerebellum and brainstem. (**A—H**) Astrovirus capsid protein (brown) in neuronal perikarya and projections (dendrites and axons) in indicated brain structures. Heatmap (yellow-low to red-high) of the neuronal virus infection burden along the neural axis is shown on the left of each image panel. Colors are based on the normalized counts of AstV+ neurons per mm^2^ of tissue area for all structures, except for Purkinje cells (PCs) in the cerebellar cortex, which is based on the number of AstV+ PC profiles per linear mm of PC layer. (**I**) Radar graph represents the entire brain clockwise from the cerebral cortex to the medulla and shows the normalized numbers of AstV+ neurons in each structure (data are presented as mean counts [red line] ± SE [gray dashed lines]). (**J** and **K**) Double immunofluorescent staining for the neuronal somatodendritic marker MAP2 (red), AstV capsid protein (green), and DAPI nuclear counterstain (blue) in the indicated brainstem structures with high neuronal infection burden. Note: (i) accumulation of aging pigment lipofuscin, which is known to be autofluorescent, produces an intense red signal in the perikarya of AstV-negative (AstV-) MAP2++ neurons and dendritic profiles and this signal colocalizes (yellow) with AstV (green) signal in infected neurons; and (ii) there is a paucity of AstV-/MAP2++ dendritic profiles and MAP2 signals are either low (MAP2+) or absent (MAP2-) in AstV+ dendritic profiles. Labeling keys used in (J and K) are provided at the bottom of the figure. Scale bars: 10 μm.

Next, we used antibody to AstV capsid protein [13,17] and performed immunohistochemistry to identify AstV-infected cells and their distribution within the human brain. The topology and morphology of AstV infected cells (Fig 1A–1H), as well as confirmatory double immunofluorescent staining for AstV capsid protein and microtubule associated protein 2 (MAP2; pan-neuronal somatodendritic marker) (Fig 1J and 1K), unambiguously demonstrated that HAstV-NIH infects exclusively neurons. Based on the normalized counts of infected neurons (see Materials and Methods), a caudal gradient in the burden of neuronal virus infection along the neural axis was evident: from a low level infection in the cerebral cortex to a high level in the cerebellum (i.e., deep cerebellar nuclei and Purkinje cells [PCs] in the cerebellar cortex) and brainstem (i.e., pontine nuclei and medullary reticular formation) (Fig 1A–1I). AstV capsid protein was present not only in the perikarya of neurons, but also in their projections (dendrites and axons) over a substantial distance from the neuronal cell body. Notably, the areas containing infected neurons also displayed a general paucity in MAP2 immunoreactivity, and somatodendritic compartments that harbored AstV capsid protein accumulations were depleted of MAP2 (Fig 1J) (compared to virus negative somatodendritic compartments, Fig 1K), suggesting disruption of integrity of the neuronal somatodendritic compartments.

Furthermore, we found AstV-infected neurons in several specific brain structures. The same structures have been reported to harbor neurons infected by another neuropathogenic virus, West Nile virus, that spreads within the CNS transsynaptically [35]. These AstV-infected structures were the basal ganglia (i.e., putamen; Fig 1B), thalamus (Fig 1D), deep cerebellar nuclei (Fig 1E), Purkinje cells in the cerebellar cortex (Fig 1F), pontine nuclei (Fig 1G and 1J), and medullary reticular formation (Fig 1H and 1K). This indicates that AstV may also spread between connected neurons transsynaptically, in both anterograde and retrograde directions.

Together, these findings demonstrate that neurons are the principal cells targeted by HAstV-NIH in the brain and that the cerebellum and brainstem carry the highest burden of infection. This is consistent with most reports that detected AstV in the CNS neurons of infected humans and animals (S1 Table). In addition, these findings suggest a transsynaptic mode of virus spread within human CNS with concurrent disruption of integrity of neuronal somatodendritic compartments.

### Fatal outcome AstV infection in the human brain is defined by disruption of neurophysiology

To understand the effect of AstV infection on the CNS at a molecular level, we performed RNA-seq and interrogated changes in gene expression in the brain of the patient with HAstV-NIH. We first used an agnostic approach that allows analysis of major coordinated shifts in the levels of gene expression from overall cumulative distribution in a particular tissue, without the need to determine the differentially expressed genes (DEGs). For this, we used the Panther statistical enrichment test (SET) [36]. SET uses unnormalized expression values (i.e., number of transcript reads > 0, for this study) for all expressed genes and returns the significantly enriched gene ontology (GO) terms for genes whose expression values were above or below the overall cumulative distribution. We designated genes whose expression levels in normal control brain were above the cumulative distribution as highly expressed and genes whose expression levels were below the cumulative distribution as suppressed. To infer about the functionality of major transcriptional shifts associated with AstV-ND, we performed SET on the unnormalized gene expression data from the brain of the patient with HAstV-NIH and from the brain of an age-matched individual without known neurological disease. For this comparison, we chose to use a normal control sample from the thalamus, which clustered together with the AstV-ND sample based on gene expression data (see Materials and Methods and S3 Fig) and was also age-matched.

**Fig 2:**
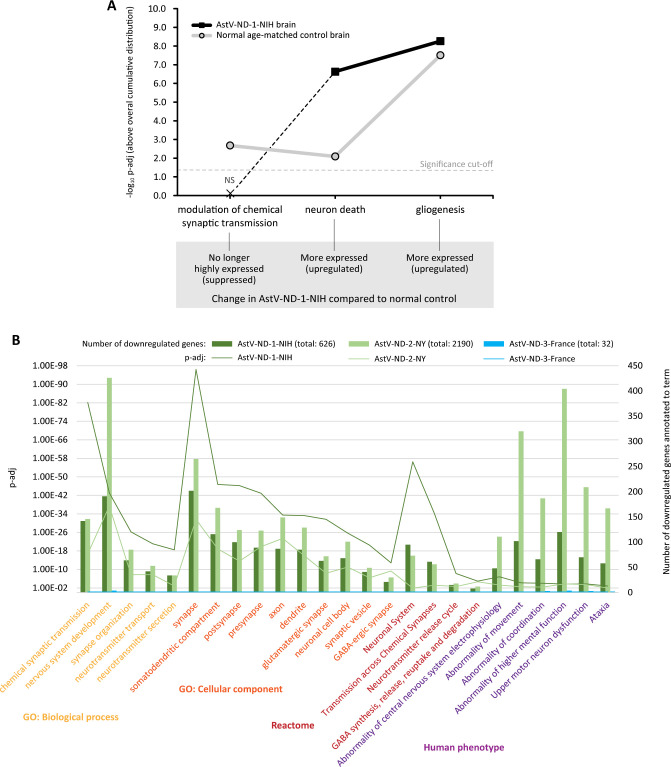
Functional genomic analysis of changes in transcriptional regulation of neurophysiology in AstV-ND. (**A**) Coordinated shifts in transcriptional regulation of brain homeostasis in AstV-ND-1-NIH compared to a normal age-matched control. Plotted are negative log_10_ FDR-adjusted p-values (-log_10_ p-adj) for major Gene Ontology (GO) terms of interest determined by SET. Dashed line indicates the significance cut-off. Inferred changes in AstV-ND-1-NIH compared to normal control for each term are indicated in the gray box. (**B**) Multi-source functional enrichment for downregulated gene expression in AstV-ND. The plot shows significantly enriched terms of interest across multiple ontologies (x-axis) with their p-adj values (left y-axis) and the number of downregulated genes annotated to each term (right y-axis). Genomic ontology sources and their corresponding terms are indicated by the same color.

Compared to a normal age-matched control, transcriptional modulation of chemical synaptic transmission in the brain of the patient with HAstV-NIH shifted below the significance cut-off value (False Discovery Rate [FDR] adjusted p value; p-adj < 0.05) (Fig 2A), indicating that genes associated with this term were no longer highly expressed. Conversely, the functional GO categories associated with the neuron death and gliogenesis shifted to higher levels of significance in the brain of the AstV-ND-1-NIH patient compared to a normal brain, indicating that these processes were upregulated.

We next asked whether the transcriptional dysregulation of neurophysiology was a common feature of human AstV-ND associated with AstVs of the VA1 genotype. Accordingly, we investigated the differential gene expression in the brain samples from three patients with AstV-ND: (i) AstV-ND-1-NIH (infected with HAstV-NIH), (ii) AstV-ND-2-NY (infected with HAstV-PS [13]), and (iii) AstV-ND-3-France (infected with HAstV-VA1/HMO-C-PA [15]). The two outside cases occurred in adolescent males with an underlying primary B cell immunodeficiency (X-linked agammaglobulinemia, XLA); one patient (AstV-ND-2-NY) died and the other (AstV-ND-3-France) survived the infection (S1 Table). Postmortem brain tissue was obtained from the two fatal cases (AstV-ND-1-NIH and AstV-ND-2-NY) and from a biopsy obtained 4.5 years after the onset of neurological symptoms in the patient who survived his infection (Ast-ND-3-France). The disease duration spanned several months to years in these three cases and while the time intervals from the onset of neurological disease to obtaining the brain samples varied, all patients had severe neurological findings when the tissue was obtained. In addition, while our RNA-seq was focused on the human transcripts, we were able to detect astrovirus sequence reads in the brain samples from all three patients with AstV-ND, but not in any of normal control brain samples, and all these astrovirus sequences were mapped to a reference VA1/HMO clade astrovirus (HAstV-VA1/HMO-C-UK1; [3]) (S2 Table). Thus, we consider these three cases of AstV-ND comparable based on the following common criteria: (i) underlying impairment of adaptive immunity (specifically, B cell immunity); (ii) neurological disease caused by AstV of VA1 genotype; and (iii) analysis of the pathogenesis in the brain at the peak of the disease.

The DEGs for each AstV-ND case were determined by comparisons to the appropriate normal controls (S3 Fig), followed by a side-by-side functional genomic analysis using a multiquery feature of gProfiler (see Materials and Methods).

Functional analysis of genes that were downregulated in the brain of three patients with AstV-ND showed that virtually every neuronal subcellular compartment was affected in the two fatal cases (AstV-ND-1-NIH and AstV-ND-2-NY), but not in the brain of the patient that survived the infection (AstV-ND-3-France). These included genes encoding both excitatory and inhibitory synapses (glutamatergic and GABA-ergic, respectively), neuronal cell bodies, dendrites, and axons (Fig 2B; GO Cellular component). This corresponded to functional impairment of the biological processes such as neurotransmission and synapse organization (Fig 2B; Biological process and Reactome). Interestingly, both AstV-ND-1-NIH and AstV-ND-2-NY also had similar significant enrichments in human disease associations, which included Human Phenotype Ontology (HP) terms describing abnormalities in electrophysiology, movement, coordination, and higher mental function (Fig 2B; Human phenotype). The complete results of functional genomic analysis of the downregulated genes in three AstV-ND cases are provided in S1 File (gProfiler Multiquery Downregulated Genes). Taken together, these results indicate that fatal AstV-ND is associated with a severe transcriptional dysregulation of neuronal cellular compartments and impairment of neurotransmission.

**Fig 3:**
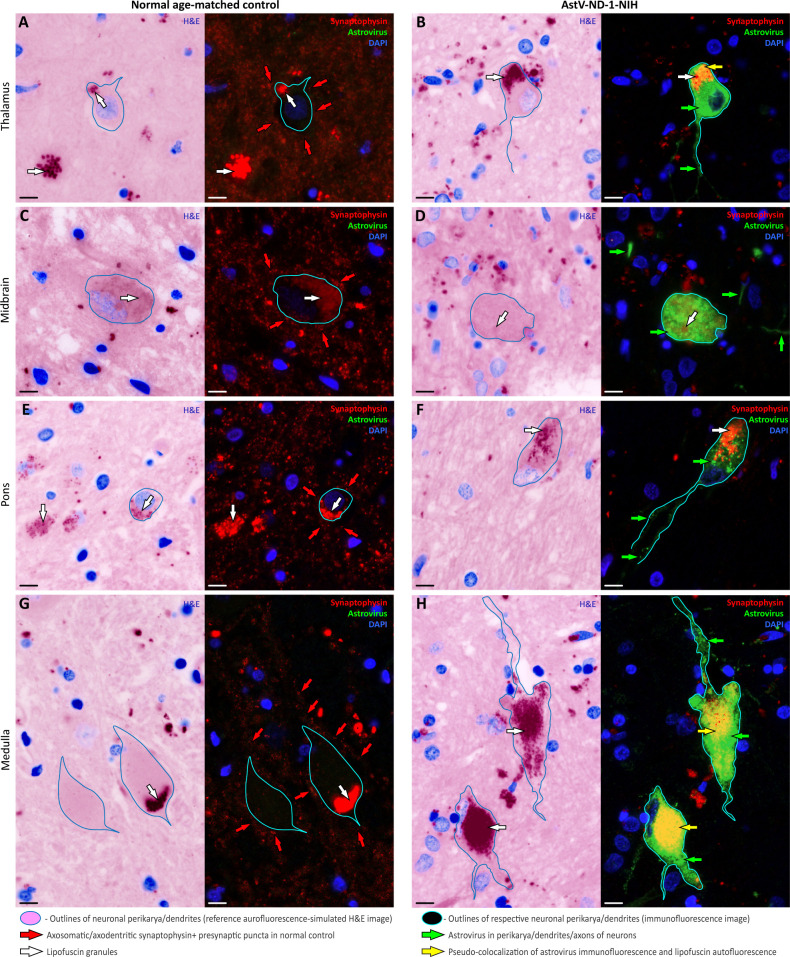
AstV replication in neurons triggers loss of their afferent innervation. (**A**—**H**) Representative images show a side-by-side comparison of the density of synaptophysin-immunoreactive presynaptic terminals in the neuropil surrounding uninfected neurons (“Normal age-matched control” column) versus astrovirus-infected neurons (“AstV-ND-1” column) in indicated brain regions. Each panel is composed of (1) an autofluorescence-simulated image that was pseudo-colored as hematoxylin-eosin (H&E) staining to serve as a topographical reference and to aid identification of intra- and extra-neuronal autofluorescent lipofuscin granules in corresponding immunofluorescent images (see Materials and Methods) and (2) immunoreactivity signals for the pan-synaptic marker synaptophysin (red), astrovirus (green), and DAPI nuclear counterstain (blue) in the corresponding tissue field. Outlines of the select neuronal perikarya/dendrites correspond to the same neurons shown in the H&E reference images and corresponding immunofluorescent images. Labeling keys used in (A—H) are provided at the bottom of the figure. Scale bars: 10 μm.

Based on the downregulation of genes associated with synapses (Fig 2B and S1 File), we examined formalin fixed paraffin-embedded (FFPE) tissue sections from AstV-ND-1-NIH by immunohistochemistry using antibodies to confirm changes in expression of proteins important for synapse organization and chemical synaptic transmission (excitatory glutamatergic and inhibitory GABA-ergic) (Table 1). Compared to a normal age-matched control, expression of synaptophysin (SYP), a protein localized to synaptic vesicles of presynaptic compartments of all types of synapses, was greatly depleted in the thalamus, midbrain, pons, and medulla of the AstV-ND-1-NIH patient (Fig 3). Importantly, synaptophysin expression was lost in the neuropil surrounding AstV-infected neurons (Fig 3B–3H). This confirms the disruption of synapse organization identified at the gene expression level (Fig 2B and Table 1 and S1 File) and indicates that AstV infection of neurons triggers loss of their afferent innervation.

**Table 1:** Analysis of disruption of neurophysiology in AstV-ND at the levels of transcriptional regulation, protein expression, and cell morphology/topology/function.

Biological process	Enriched gene ontology terms	Protein marker expression	Cellular morphology/topology/function	Representative images
**Disruption of synapse organization**	synapse organization (GO:0050808) synapse (GO:0045202) presynapse (GO:0098793) synaptic vesicle (GO:0008021) neurotransmitter secretion (GO:0007269) Neurotransmitter release cycle (REAC:R-HAS-112310) Abnormality of CNS electrophysiology (HP:0030178) Upper motor neuron dysfunction (HP:0002493)	SYP ↓ (Expressed in synaptic vesicles; pan presynaptic protein marker)	- decrease in SYP in the neuropil surrounding AstV-infected neurons; - disruption of structural integrity of presynaptic compartments; - disruption of afferent innervation of infected neurons	Fig 3B–3H
**Disruption of excitatory synaptic transmission**	chemical synaptic transmission (GO:0007268) glutamatergic synapse (GO:0098978) presynapse (GO:0098793)	VGLUT1 ↓ (Expressed in presynaptic compartments of glutamatergic synapses)	- decrease of VGLUT1 presynaptic puncta in infected brain structures; - disruption of structural integrity of presynaptic compartments of glutamatergic synapses; - disruption of excitatory afferent innervation of infected neurons	Fig 4B
**Disruption of inhibitory synaptic transmission**	chemical synaptic transmission (GO:0007268) GABA-ergic synapse (GO:0098982) GABA synthesis, release, reuptake, and degradation (REAC:R-HAS-888590) presynapse (GO:0098793)	VGAT ↓ (Expressed in presynaptic compartments of GABA-ergic synapses)	- decrease of VGAT presynaptic puncta in infected brain structures; - disruption of structural integrity of presynaptic compartments of GABA-ergic synapses; - disruption of inhibitory afferent innervation of infected neurons	Fig 4D
**Disruption of integrity of somatodendritic compartments**	somatodendritic compartment (GO:0036477) dendrite (GO:0030425) neuronal cell body (GO:0043025)	MAP2 ↓ (Expressed in somatodendritic com-partments of neurons)	- decrease in MAP2 in infected brain structures; - disruption of structural integrity of somatodendritic compartments of neurons	Figs 1J, 1K and 5

↓—decreased compared to normal control. SYP, synaptophysin. VGLUT1, vesicular glutamate transporter 1. VGAT, vesicular GABA transporter. GABA, gamma-aminobutyric acid. MAP2, microtubule associated protein 2.

**Fig 4:**
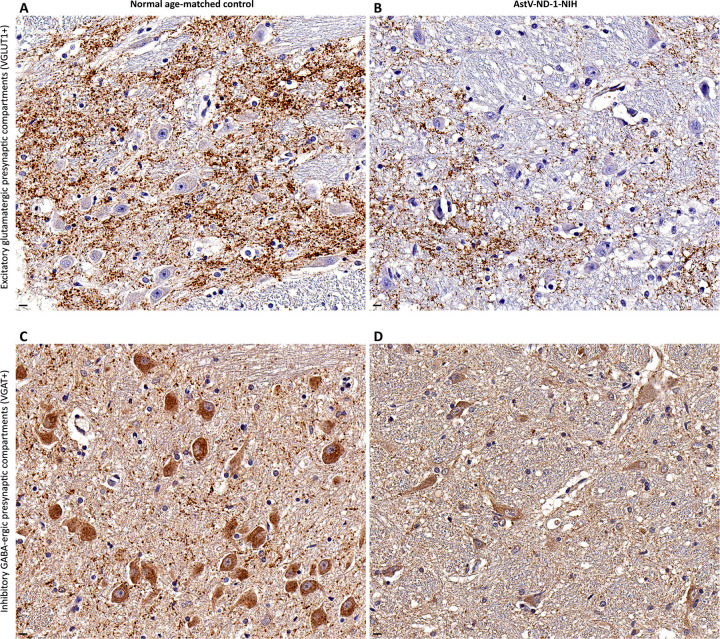
AstV infection is associated with disruption of both excitatory and inhibitory neurotransmission. (**A**—**D**) Representative images show a side-by-side comparison of the density (brown immunoreactivity) of presynaptic compartments of excitatory glutamatergic (vesicular glutamate transporter 1 [VGLUT1]+) synapses (A and B) and inhibitory GABA-ergic (vesicular GABA transporter [VGAT]+) synapses (C and D) in the brainstem of a normal age-matched control subject (A and C) and in the brainstem of AstV-ND-1-NIH (B and D). Note an extensive loss of the excitatory (B) and inhibitory (D) presynaptic puncta in AstV-ND-1-NIH, compared to a normal age-matched control (A and C, respectively). Scale bars: 10 μm.

Expression of the vesicular glutamate transporter 1 (a protein localized to excitatory glutamatergic synapses) was markedly reduced (Fig 4A and 4B). This confirms the disruption of excitatory synaptic transmission identified at the gene expression level (Fig 2B and Table 1 and S1 File) and indicates that AstV infection of neurons disturbs excitatory afferent innervation of infected neurons. Expression of the vesicular GABA transporter (a protein localized to inhibitory GABA-ergic synapses) was also markedly reduced (Fig 4C and 4D), confirming the transcriptional dysregulation of inhibitory synaptic transmission and inhibitory afferent innervation of infected neurons (Fig 2B and Table 1 and S1 File).

**Fig 5:**
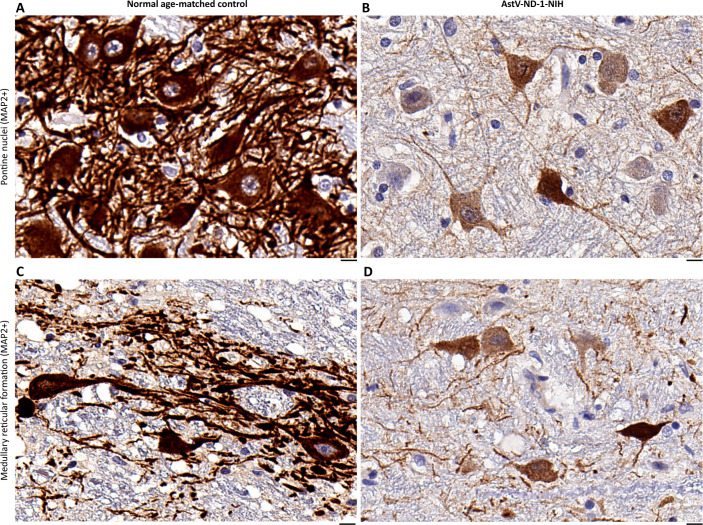
AstV infection is associated with disruption of structural integrity of neuronal somatodendritic compartments. (**A**—**D**) Representative images show a side-by-side comparison of the MAP2 immunoreactivity (brown) in somatodendritic compartments of neurons in the pontine nuclei (A and B) and medullary reticular formation (C and D) in the brainstem of a normal age-matched control subject (A and C) and in the brainstem of AstV-ND-1-NIH (B and D). Note an extensive loss of the MAP2 immunoreactivity in AstV-ND-1-NIH, compared to a normal age-matched control. Scale bars: 10 μm.

Since we observed depletion of MAP2 in the somatodendritic profiles that harbored AstV capsid protein by a double fluorescent immunostaining (Fig 1J and 1K) and our functional genomic analysis also revealed downregulation of genes associated with the neuronal somatodendritic compartments (Fig 2B and S1 File), we further examined changes in MAP2 expression at the protein level in the brainstem of AstV-ND-1-NIH patient by immunohistochemistry, in comparison to a normal age-matched control. As anticipated, MAP2 expression was markedly reduced in the neuronal somatodendritic compartments of AstV-ND-1-NIH compared to a normal age-matched control (Fig 5 and Table 1 and S1 File). This confirms that AstV infection of neurons in the brain of AstV-ND-1-NIH disrupted the integrity and function of neuronal somatodendritic compartments.

Taken together, these results validate the gene expression data by demonstrating the loss of expression of critical proteins functioning in the synaptic and somatodendritic compartments of neurons. The net impact of AstV infection of neurons was disruption of synaptic and somatodendritic integrity, loss of afferent innervation of infected neurons, and impairment of both excitatory and inhibitory neurotransmission. Impairment of these essential neural functions may have contributed to the fatal outcome in AstV-ND-1-NIH, and possibly in AstV-ND-2-NY.

### Innate immune responses drive host defense to AstV infection in the brain of patients with impaired adaptive immunity

**Fig 6:**
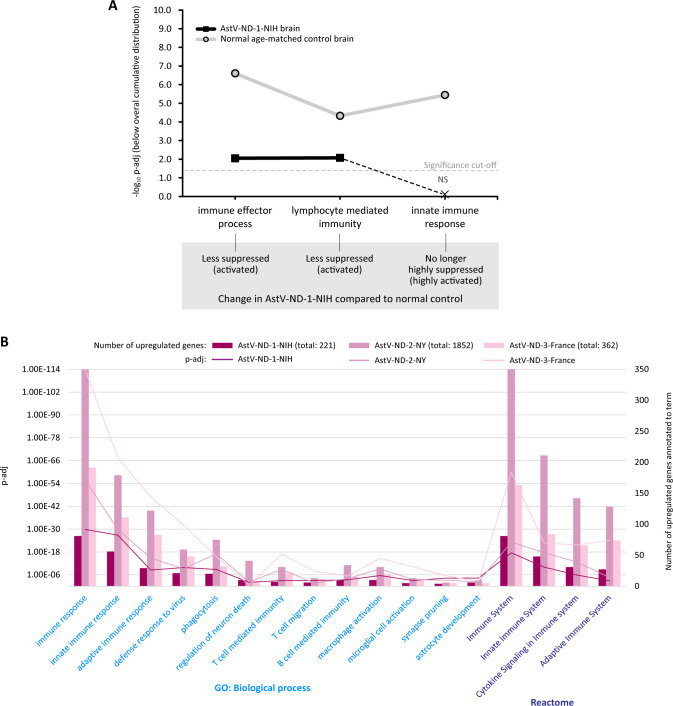
Functional genomic analysis of transcriptional regulation of immune responses in the brain of patients with AstV-ND. **(A)** Coordinated shifts in transcriptional regulation of immune processes in the brain of AstV-ND-1-NIH compared to a normal age-matched control. Plotted are negative log_10_ FDR-adjusted p-values (-log_10_ p-adj) for major Gene Ontology (GO) terms of interest determined by SET. Dashed line indicates the significance cut-off. Inferred changes in AstV-ND-1-NIH compared to normal control for each term are indicated in the gray box. (**B**) Multi-source functional enrichment for upregulated gene expression in AstV-ND. The plot shows significantly enriched terms of interest across multiple ontologies (x-axis) with their p-adj values (left y-axis) and the number of upregulated genes annotated to each term (right y-axis). Genomic ontology sources and their corresponding terms are indicated by the same color.

To gain insight into host defense responses to AstV infection in the brain of patients with impaired adaptive immunity, we first performed SET on unnormalized gene expression data from brain samples of AstV-ND-1-NIH and a normal individual without known neurological disease. We focused on three major gene ontology terms critical for host defense to viral infections of the CNS: immune effector processes, innate immune response, and lymphocyte mediated immunity. As expected, these immune processes were relatively suppressed (below overall cumulative distribution) in the normal brain (Fig 6A). In the brain of AstV-ND-1-NIH, these immune processes were either less suppressed (immune effector process and lymphocyte mediated immunity) or no longer suppressed (innate immune response) (Fig 6A).

Next, we determined the upregulated genes in brain tissue from the three cases of AstV-ND (AstV-ND-1-NIH, AstV-ND-2-NY, and AstV-ND-3-France) by comparing them with appropriate normal controls (S3 Fig). An analysis of the upregulated genes using gProfiler showed a trend in the level of functional enrichment of specific terms related to immune responses that applied to all three patients (Fig 6B). Notably, the patient that survived AstV infection (AstV-ND-3-France) had the highest functional enrichment of immune responses. Of all the specific immune GO and Reactome terms analyzed, the innate immune response was most significantly upregulated, compared to the adaptive immune response, in all three patients with AstV-ND (Fig 6B). Associated with the innate immune response were genes related to the defense response to virus, phagocytosis, macrophage/microglial activation, astrocytic response, and synapse pruning. There was considerably less transcriptionally activated adaptive immune response which included terms related to B-cell and T-cell mediated immunity, and T cell migration. In addition, brain tissue from all three patients with AstV-ND had a low-level transcriptional upregulation of neuron death. The complete results of functional genomic analysis of the upregulated genes in three AstV-ND cases are provided in S2 File (gProfiler Multiquery Upregulated Genes). Taken together, the transcriptome results reveal that innate immune responses, including phagocytosis, macrophage activation, and astrocytic hypertrophy are the most important components of the CNS host defense response to AstV infection in patients with a premorbid impairment in adaptive immunity.

**Fig 7:**
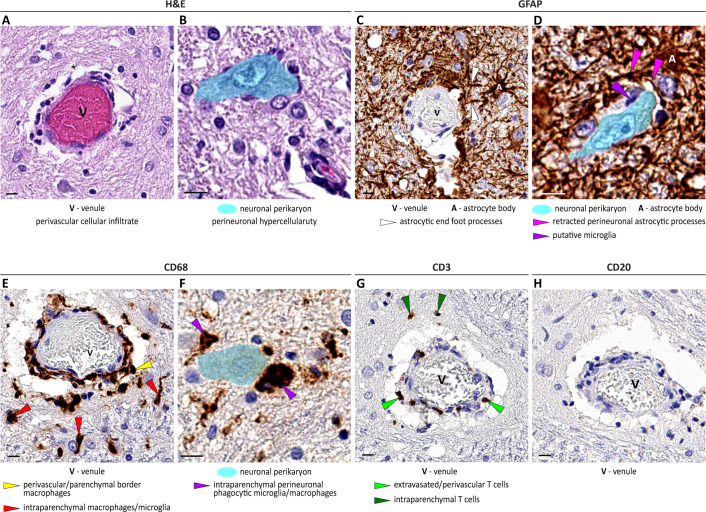
Cellular responses to AstV infection in the brainstem of an immunocompromised adult. **(A—H)** Representative images show perivascular (A, C, E, G, and H) and perineuronal (B, D, and F) tissue in adjacent sections of the medulla that illustrate: (1) perivascular cellular infiltrate (A) and perineuronal hypercellularity (B) (hematoxylin and eosin [H&E]); (2) moderate astrocytosis (C and D; glial fibrillary acidic protein [GFAP]; brown) with (i) hypertrophy of astrocytic somata and perivascular end foot processes (C) and (ii) retraction of perineuronal astrocytic processes and a cell with microglial morphology (putative microglia) that is in close apposition to the neuronal membrane (D); (3) CD68+ perivascular/parenchymal border macrophages and intraparenchymal microglia/macrophages (E; brown); (4) intraparenchymal/perineuronal phagocytic microglia/macrophages (F; brown); (5) infiltrating (extravasated/perivascular/intraparenchymal) CD3+ T cells (G; brown); and (6) absence of infiltration by CD20+ B cells (H). Scale bars: 10 μm.

To confirm the transcriptome results at the protein expression and cell morphology/topology/function levels, we examined tissue sections from AstV-ND-1-NIH using immunohistochemistry and evaluated reactive astrocytosis, microglia/macrophage activation and migration, and lymphocytic activation, migration, and infiltration (Table 2). Staining with hematoxylin and eosin showed that the brainstem, which had the highest burden of AstV infected neurons (Fig 1G–1K), had perivascular cellular infiltration (Fig 7A) and perineuronal hypercellularity (Fig 7B).

**Table 2:** Analysis of immune responses associated with AstV-ND at the levels of transcriptional regulation, protein expression, and cell morphology/topology/function.

Biological process	Enriched gene ontology terms	Protein marker expression	Cellular morphology/topology/function	Representative images
**Reactive astrocytosis**	glial cell activation (GO:0061900) astrocyte development (GO:0014002) astrocyte activation (GO:0048143) astrocyte differentiation (GO:0048708)	GFAP ↑ (increased expression in reactive astrocytes)	***Astrocytes***: hypertrophy of somata of perivascular and perineuronal astrocytes and their processes	Fig 7C and 7D
**Microglia/macrophage activation and migration**	innate immune response (GO:0045087) Innate Immune System (REAC:R-HSA-168249) glial cell activation (GO:0061900) macrophage activation (GO:0042116) microglial cell activation (GO:0001774) phagocytosis (GO:0006909) synapse pruning (GO:0098883) complement-mediated synapse pruning (GO:0150062)	CD68 ↑ (expressed by both activated macrophages and microglial cells)	***Macrophages*:** hypertrophy of somata; perivascular, parenchymal border, intraparenchymal, and perineuronal topology; phagocytic activity ***Microglial cells***: hypertrophy of somata and processes; neuron-centripetal migration; perineuronal topology; phagocytic activity	Fig 7E and 7F
**Lymphocytic activation, migration, and infiltration**	adaptive immune response (GO:0002250) Adaptive Immune System (REAC:R-HSA-1280218) lymphocyte mediated immunity (GO:0002449) lymphocyte activation (GO:0046649) T cell activation (GO:0042110) T cell migration (GO:0072678) T cell mediated immunity (GO:0002456) B cell mediated immunity (GO:0019724)	CD3 ↑ (pan T cell protein marker)	***T cells***: minimal leptomeningeal infiltration; minimal perivascular infiltration; restricted parenchymal migration	Fig 7G
		CD20—negative (pan B cell protein marker)	***B cells***: No leptomeningeal, perivascular, or parenchymal infiltration	Fig 7H

↑—increased compared to normal control. GFAP, glial fibrillary acidic protein. CD68, lysosomal-associated membrane protein. GO, Gene Ontology. REAC, Reactome pathways.

Expression of glial fibrillary acidic protein, a marker for astrocyte activation associated with neuronal injury, was increased in infected brain tissue along with hypertrophy of the soma of perivascular (Fig 7C) and perineuronal (Fig 7D) astrocytes and their processes, consistent with reactive astrocytosis [37]. In addition, the perineuronal astrocytic processes appeared to be partially retracted from the neuronal perikarya, with the resulting space occupied by migrated cells with the morphology of microglia (Fig 7D). Taken together, these findings are consistent with the enrichment in GO terms related to the glial cell activation and astrocyte development/activation/differentiation (Fig 6B and Table 2 and S2 File)

The lysosomal-associated membrane protein CD68, a protein marker of activated phagocytic macrophages and microglia, was detected in numerous cells in the perivascular, parenchymal border, intraparenchymal, and perineuronal locations in AstV-ND-1-NIH (Fig 7E and 7F). Notably, activated/reactive microglial cells displayed neuron-centripetal migration (likely beyond the retracted perineuronal astrocytic processes) and perineuronal topology (Fig 7F; compare to Fig 7D). These changes in morphology and topology of macrophages and microglial cells corresponded to the significantly enriched GO terms related to innate immunity, activation of macrophages and microglial cells, phagocytosis, and synapse pruning (Fig 6B and Table 2 and S2 File).

Minimal numbers of CD3+ T cells were detected in leptomeningeal and perivascular locations, with very restricted migration and infiltration of the adjacent perivascular parenchyma (Fig 7G). No CD20+ B cells were detected in leptomeningeal, perivascular, or parenchymal sites (Fig 7H). These findings are consistent with the low ranked transcriptional activation of the adaptive immune response, compared with the robust activation of innate immunity (Fig 6B and Table 2 and S2 File). Taken together, these results indicate that in the setting of an impaired adaptive immune response, brain cellular responses to AstV infection heavily rely on the innate immune responses of resident CNS glial cells (astrocytes and microglia) and activated perivascular/parenchymal border macrophages.

## Discussion

In this study, we investigated the neuropathogenesis of AstV-ND based on the changes in gene expression in three patients with the disease and confirmed the findings using immunohistochemistry in several regions of the brain of one of these patients. We demonstrate that neurons are the principal cells targeted by a neuropathogenic VA1 AstV in the human brain and localized AstV capsid protein to the neuronal perikarya, dendrites and axons. These findings indicate that AstV, similar to many other neuropathogenic viruses [38], can spread along neuronal projections and possibly transsynaptically between connected neurons. Intriguingly, we found AstV capsid protein in the same brain structures (i.e., thalamus, deep cerebellar nuclei, Purkinje cells, and pontine nuclei) that are preferentially infected in primates by West Nile virus [35]. This indicates that AstV may spread between connected neurons transsynaptically in a retrograde manner (e.g., thalamus to deep cerebellar nuclei to Purkinje cells). Therefore, as has been shown for West Nile virus infection [9], transsynaptic propagation of AstV infection within the CNS may result in profound neurophysiological impairment.

The gradient of neuronal astrovirus infection in the human brain intensifies caudally with the major impact on the brainstem, followed by the cerebellum. A similar caudal gradient of neuronal vulnerability to AstV infection has been seen in the CNS of other mammals (S1 Table). Neuropathogenic viruses can either induce death of neurons or cause noncytolytic neuronal infections [7,39]. The virus infection and ensuing immune responses may interfere with neuronal signaling and disturb synaptic activity [7, 9,40–42]. Indeed, in this study, both brain transcriptome and immunohistochemistry demonstrated the impact of AstV infection on neurophysiology. We found that AstV infection of neurons in the human brain impacts neurophysiology by disrupting synaptic integrity, triggering a loss of afferent innervation related to infected neurons, and impairing both excitatory and inhibitory neurotransmission. Importantly, based on changes in gene expression, this functional impairment was an important attribute of changes in the brain homeostasis in fatal cases, but not in a surviving patient with AstV-ND. This suggests that the outcome of AstV-ND in immunocompromised patients depends on the degree to which essential neuronal functions are impaired. Since studies of postmortem brain tissue from humans who died from other viral CNS infections also showed downregulation of expression of neural genes [43,44] and synaptic degeneration and loss [45–49], these neuropathological processes may be common to many fatal CNS infections in humans.

The human CNS has barriers to protect itself from an invasion by various pathogens (including viruses) that otherwise would be detrimental to neural function and could be life-threatening. Viremia ensues when virus replication in the peripheral tissue reaches a threshold such that it can no longer be contained, and the virus enters the bloodstream. Viremia facilitates virus spread to various tissues (including peripheral nerves) and increases the probability of virus invasion of the CNS. Indeed, viremia is a major neuropathogenic determinant for viral CNS infection [8]. However, immunocompetent hosts with intact innate and adaptive immunity are usually able to contain virus replication in the periphery, reduce the level of viremia, and prevent virus invasion of the CNS. In contrast, immunocompromised persons who fail to mount successful innate and/or adaptive antiviral immune responses in the periphery may develop devastating neurological disorders associated with viral neuroinvasion. Viremia has indeed been reported in association with AstV infection of the CNS [14,17]. Immunocompromised persons may have primary immunodeficiencies (such as genetic disorders that impair the innate and/or adaptive immune response), secondary immunodeficiencies (including recipients of immune-cell depleting and/or immunomodulatory therapies or those with AIDS), or be at the extremes of age (including infants with immature immune systems or the elderly with immunosenescence) [1].

Underlying conditions in patients with AstV-ND in this study included both primary and secondary immunodeficiencies (S1 Table) predominantly affecting B cell immunity (XLA or lymphoma/leukemia with B-cell depleting and immunosuppressive treatments). Adaptive immunity, including B cell activity, is important to contain viral infections in the periphery to prevent invasion of the CNS, as well as to promote cytolytic and non-cytolytic virus clearance in the CNS (reviewed in [34,50]). Our findings at the transcriptional, protein expression, and cell morphology level show that defective adaptive immunity in patients with AstV-ND impairs responses to astrovirus infection within the CNS. Nevertheless, we detected robust changes in gene expression in the brain that were associated with activation of innate immune responses to astrovirus infection in patients with fatal AstV-ND and in a patient who survived the infection. These findings were corroborated by immunohistochemical analysis of CNS tissues, which showed activation of astrocytes, microglia, and macrophages. As expected, deficiency in B cells whether due to XLA [51] or hematologic malignancy with B-cell depleting and immunosuppressive treatments, results in the absence of B cell/plasma cell migration to the brain to control astrovirus infection. Indeed, similar to other cases of CNS astrovirus infections in immunocompromised persons (summarized in S1 Table), we found only minimal lymphocytic infiltration of the brain, which was composed predominantly of T cells. In contrast, astrovirus infection of the CNS in animals is often associated with prominent perivascular and leptomeningeal lymphocytic infiltration (S1 Table) with admixed plasma cells [25]. Interestingly, astrocytic retraction of their perineuronal processes appeared to play a supportive role by facilitating access for activated microglia/macrophages to position themselves close to infected neurons. On the other hand, hypertrophy of perivascular astrocytic end feet may limit excessive parenchymal infiltration by peripheral immune cells from perivascular spaces. Taken together, these findings suggest that in the absence of effective adaptive immunity, an overreliance on innate immune mechanisms, such as phagocytic activity of microglia and macrophages directed to clear already damaged cells and their debris, may lead to excessive damage to the host.

One limitation of this study is a small sample size, which included brain samples from only three cases of human AstV-ND that we were able to obtain. However, considering that only 14 cases of AstV-ND have been reported to date, with the neuropathological findings available only for 7 single cases (S1 Table) [13–24], our study provides the largest and most comprehensive comparative data of the neuropathogenesis of AstV-ND in humans. Another limitation is that a sufficient number of the brain structures to perform the molecular pathology analyses and to validate transcriptomic findings at the protein level was only available from one patient; however, the transcriptomics results on the impaired neurophysiology were consistent between the two fatal cases, and the brain host defense responses to AstV infection were consistent in all three cases.

With the increasing use of immunosuppression for a variety of disorders (e.g., autoimmune disease, organ/stem cell transplantation, and cancer) predisposing to opportunistic viral infections of the CNS, as well as the emergence or reemergence of neurotropic viruses (e.g., enterovirus D68, West Nile, Chikungunya, Zika, Hendra, and Nipah viruses) [4,52] the frequency of viral encephalitides is expected to increase in the coming years. In addition, global warming may contribute to spread of viral vectors in new areas that can increase the prevalence of arthropod borne viruses to new populations [53]. These changes will continue to present major challenges for the diagnosis and treatment of these diseases as well as for public health measures to limit the spread of these viruses. Since effective treatments do not exist for most opportunistic viral infections of the CNS, the only options are the reversal of immunosuppression or the use of immunomodulatory agents such as virus-specific T cells [54] or checkpoint inhibitors [55]. However, enhancing immune responses may increase the risk of worsening the underlying condition [52] or induce the immune reconstitution inflammatory syndrome [54] which can result in enhanced neuropathology. Thus, a better understanding of the pathophysiology of opportunistic viral infections of the CNS, including astrovirus encephalitis, is needed to limit neurologic damage associated with these diseases and to develop new therapeutic approaches.

## Materials and methods

### Ethics statement

Consent for research studies was obtained for use of leftover brain tissue from AstV-ND-3-France from the patient’s parents; brain tissue was obtained postmortem from AstV-ND-1-NIH and AstV-ND-2-NY and as such is not considered human subjects research.

### Brain tissue samples

For RNA-seq, frozen brain tissue samples from three patients with AstV-ND were used: (i) AstV-ND-1-NIH (unknown brain site; 58-year-old man; see case description below); (ii) AstV-ND-2-NY (brainstem; a 15-year-old boy with X-linked agammaglobulinemia [XLA]) [13]; and (iii) AstV-ND-3-France (frontal cortex; a 14-year-old boy with XLA) [15]. Frozen brain tissue samples from subjects (ages 14 to 58-years old) without known neurological disease were used as normal controls (frontal cortex [n = 2]; thalamus (n = 1); brainstem [n = 1]; medulla [n = 1]; and cerebellum [n = 1]). These normal tissue samples were obtained from the Human Brain Collection Core, Intramural Research Program, NIMH (http://www.nimh.nih.gov/hbcc). Formalin-fixed paraffin-embedded (FFPE) brain tissue sections from AstV-ND-1-NIH case were used for histopathological analysis (hematoxylin-eosin [H&E] staining), brightfield colorimetric immunohistochemistry, and double immunofluorescent staining. FFPE sections from an age-matched subject without known neurological disease were used as normal controls.

### AstV-ND-1-NIH case description

A 58-year-old man presented with altered mental status after receiving a double unit umbilical cord blood transplant for lymphoma. The patient was diagnosed with follicular lymphoma, was treated with rituximab, cyclophosphamide, doxorubicin, vincristine, and prednisone therapy for 6 cycles and underwent remission. He was diagnosed with recurrent disease three years later and was treated with radiation therapy. Four months later biopsy of a new soft tissue mass showed diffuse large B cell lymphoma compatible with transformation of follicular lymphoma, and he received three cycles of rituximab, dexamethasone, cytarabine, and cisplatin therapy. He received conditioning with cyclophosphamide, thiotepa, fludarabine, and total body irradiation (400 cGy) and underwent a double cord blood stem cell transplant. He received cyclosporine and mycophenolate mofetil for graft-versus-host disease prophylaxis. He engrafted with 100% donor cells but developed gastrointestinal graft-versus-host disease and was treated with methylprednisolone and budesonide and his cyclosporine and mycophenolate mofetil were continued. One month after transplant, the patient presented with lower extremity sensory changes and weakness with ascending neuropathy, ataxia, unstable gait, and fatigue. An MRI of the brain and cervical spine were unremarkable, and an EMG showed a sensory motor neuropathy. The cerebrospinal fluid (CSF) showed 5 white blood cells/ml, protein 55 mg/dL, glucose 57 mg/dL and was negative for human herpesvirus 6, varicella-zoster virus, herpes simplex virus, JC virus, and cytomegalovirus. Cytology showed atypical lymphocytes. A paraneoplastic panel was negative and oligoclonal bands were not detected. Three months after his neurologic changes began, a PET scan of brain showed a mildly abnormal thalamus with increase uptake, and one week later, an MRI showed a new fluid attenuated inversion recovery hyperintensity with restricted diffusion in the right midbrain, both cerebellar peduncles, and right lateral pons. An EEG showed background slowing, and the CSF showed protein 48 mg/dL, glucose 65 mg/dL, white blood cell count 5 cells/ml with 68% lymphocytes, and many atypical lymphocytes and was again with negative for human herpesvirus 6, JC virus, and cytomegalovirus. He was treated with acyclovir and foscarnet followed by rituximab and solumedrol. He developed progressive mental status changes, weakness, extra-pyramidal signs, and agitation. An EEG showed generalized slowing and an EMG showed a sensorimotor polyneuropathy involving the bilateral lower extremities that was ascending. CSF showed elevated protein and lack of pleocytosis, and he was treated with high dose intravenous immunoglobulin and steroids for presumptive variant Guillain-Barre. A sural nerve biopsy showed nerve damage. He received an additional course of rituximab and died seven months after transplant. At autopsy, the diagnosis was a multifocal leukoencephalopathy of unknown etiology.

### Astrovirus RNA detection

#### Virus microarray

RNA was isolated from frozen brain tissue sample from AstV-ND-1-NIH case and hybridized to a microarray containing >3000 viral probes for viruses spanning 31 virus families known to infect vertebrates and >19,000 probes for endogenous genes. The virus microarray and bioinformatics analysis were performed as previously described [56]. An average probe intensity from control brain tissue was used to define the background threshold (average probe intensity plus three standard deviations).

#### PCR

RNA was isolated from frozen brain tissue sample from AstV-ND-1-NIH case and cDNA was generated using a Superscript First-Strand Synthesis System for RT-PCR using random hexamers (Invitrogen). A first round of PCR was performed using primers panAV-F11 (5′-GARTTYGATTGGRCKCGKTAYGA-3′), panAV-F12 (5′-GARTTYGATTGGRCKAGGTAYGA-3′) and panAV-R1 (5′-GGYTTKACCCACATICCRAA-3′), followed by a second round of PCR with primers panAV-F21 (5′-CGKTAYGATGGKACKATICC-3′), panAV-F22 (5′-AGGTAYGATGGKACKATICC-3′) and panAV-R1 as previously described [57].

#### In situ hybridization

RNA was isolated from frozen brain tissue sample from AstV-ND-1-NIH case and cDNA was generated using a Superscript First-Strand Synthesis System for RT-PCR using random hexamers (Invitrogen). Astrovirus capsid primers, Astrocap for 5- CGC GCG GAT CCA CCA TGG GGG GAT GGT GGT TTG TCA AG -3 and Astrocap rev 5- CGC GCG TCT AGA CTC GGC GTG GCC TCG GCG CAA -3 yielded a 1,227 bp band, which was cloned into pXLE42·V5 tag vector at the Bam HI and XbaI sites. After confirming the astrovirus capsid sequence, the negative strand was used instilling a 19-point bp change for probe creation, identical to the patient’s bp changes. Sections from the AstV-ND-1 patient’s brain and a control human brain were mounted onto microscope slides and hybridized with either astrovirus or actin probes using a QuantiGene (R superscript circularized) ViewRNA ISH Tissue 2-Plex Assay kit (Affymetrix Cat No. QVT0012). The accession number for the HAstV-NIH astrovirus sequence in Genbank is OP293097.2 (https://www.ncbi.nlm.nih.gov/nuccore/OP293097).

### Phylogenetic analysis

A BLAST [58] search of the NCBI protein sequence database for homologs to the HAstV-NIH capsid precursor protein sequence was performed and the top 250 hits were retrieved. These sequences were filtered to remove partial sequences resulting in 225 mammalian astrovirus capsid protein sequences. Four reference, avian astrovirus protein sequences were identified and added to this data to serve as an outgroup. A multiple sequence alignment of these data was performed using the MAFFT program [59]. Two different algorithms were used to calculate phylogenetic trees from this protein multiple sequence alignment. First, a maximum-likelihood analysis was performed using the IQtree v1.6.12 software [60] using the ModelFinder [61] option to determine the best fit substitution model. Support for individual clades was assessed using 1000 bootstrap replicates of the UFBoot ultrafast bootstrap algorithm [62]. A Bayesian phylogenetic analysis also was performed. This analysis used the MrBayes v3.2.5 software [63]. A single chain analysis running for 8,000,000 generations with sampling every 800 generations was performed. The program evaluated the best fit substitution model and chose the WAG [64] model with posterior probability = 1.0. All convergence criteria were met at the end of this analysis. No significant difference was found between the two phylogenies so the IQtree phylogeny was used going forward.

### Gene expression analysis

Tissue samples were homogenized for 40 sec in lysing matrix D tubes (MP Biomedicals, Santa Ana, CA) containing 1 ml Trizol (Thermofisher Scientific, Waltham, MA) in a FastPrep FP 120 instrument (MP Biomedicals) at a speed of 6.0 meters per second. Homogenized Trizol lysate was combined 1-Bromo-3-chloropropane (MilliporeSigma, St. Louis, MO), mixed, and centrifuged at 16,000 x g for 15 min at 4°C. RNA containing aqueous phase was collected from each sample and passed through a Qiashredder column (Qiagen, Valencia, CA) at 21,000 x g for 2 min to homogenize any remaining genomic DNA in the aqueous phase. The aqueous phase was combined with an equal amount of RLT lysis buffer (Qiagen, Valencia, CA) with 1% beta-mercaptoethanol (MilliporeSigma, St. Louis, MO), and RNA was extracted using Qiagen AllPrep DNA/RNA mini columns (Valencia, CA). The RNA yield ranged from 860 ng to 12.4 μg. RNA quality was determined by spectrophotometry at 260 nm and 280 nm and by fluorescence capillary assay (RNA 6000 Pico kit, Agilent Technologies, Santa Clara, Ca). RNA integrity numbers ranged from 2.3 to 6.2.

A Truseq Stranded mRNA-Seq Sample Preparation Kit (Illumina) was used to synthesize cDNA and generate sequencing ready libraries following the manufacturer’s protocol. Due to RNA degradation and low RNA integrity values, the fragmentation time was reduced from eight to six min. Library quality and size distribution was assessed on a BioAnalyzer DNA 1000 chip (Agilent Technologies), and concentrations were determined using the Kapa SYBR FAST Universal qPCR kit for Illumina sequencing (Roche). Paired-end 75 cycle sequencing was completed using two Mid Output 150 cycle kits on the NextSeq 550 (Illumina).

Raw reads were trimmed of adapter sequence using cutadapt (https://cutadapt.readthedocs.io/en/stable/). The remaining reads were then filtered for low quality bases and low quality reads using the FASTX-Toolkit (http://hannonlab.cshl.edu/fastx_toolkit/). The remaining reads were mapped to the GRCh38 genome, using HISAT2. Reads mapping to genes were counted using htseq-count. Differential expression analysis was performed using the Bioconductor package DESeq2 along with generation of the sample heatmap using the pheatmap package with default parameters (S3 Fig).

Clustering analysis of the transcriptomes from the three tissues from patients with AstV-ND with brain tissues from normal controls (thalamus [n = 1], brainstem [n = 2], and frontal cortex [n = 2]) showed the following: (i) AstV-ND-2-NY [brainstem] transcripts clustered with the brainstem from two age-matched normal controls; AstV-ND-3-France [frontal cortex] clustered with the frontal cortex from two age-matched normal controls, validating this approach (S3 Fig). AstV-ND-1-NIH (brain tissue from unknown site) clustered with the thalamus from one age-matched normal control. DEGs were identified by comparing AstV-ND samples to normal control samples as shown on the right side of S3 Fig. Fold changes (FC) ≤ -2 and ≥ 2.0 and false discovery rate (FDR) adjusted p values (p adj) < 0.05 were used as cut-offs to define the significantly DEGs for subsequent functional genomic analyses.

Viral reads were aligned to the reference astrovirus HAstV-VA1/HMO-C-UK1 [3] (Accession number KM358468.1). Coverage (S2 Table) was generated using samtools coverage (http://www.htslib.org/doc/samtools-coverage.html).

The metadata and normalized data files have been deposited in the GEO database (https://www.ncbi.nlm.nih.gov/geo/query/acc.cgi?acc=GSE201384).

### Genomic analyses

Functional enrichment analyses of gene expression data were performed using the PANTHER statistical enrichment test (SET) and gProfiler as previously described [9]. A multi-query multi-source gProfiler platform [65] was used for simultaneous comparative functional genomic analyses of differential gene expression in three AstV-ND cases used in this study. The following significance thresholds were used for multiple testing correction: g:SCS threshold and Benjamini-Hochberg FDR (https://biit.cs.ut.ee/gprofiler/page/docs#significance_threhshold).

### Histology and Immunohistochemistry

FFPE brain tissue sections were stained with hematoxylin and eosin (H&E) or processed for immunohistochemistry. Brightfield colorimetric immunohistochemistry was performed using Bond RX (Leica Biosystems) according to manufacturer protocols. Diaminobenzidine was used for colorimetric detection (brown) and hematoxylin was used for counterstaining. The following primary antibodies were used for brightfield colorimetric immunohistochemistry: antibody against astrovirus capsid protein [13,17] (rabbit polyclonal; 1:500), anti-CD68 (mouse monoclonal [KP1]; Biocare Medical; 1:50), anti-GFAP (rabbit polyclonal; Abcam; 1:250), anti-CD3 (rat monoclonal [Clone 12]; AbD Serotec; 1:600); anti-CD20 (mouse monoclonal [Clone L26]; Agilent; 1:200); anti-VGLUT1 (rabbit polyclonal; Synaptic Systems; 1:300), and anti-VGAT (rabbit polyclonal; Synaptic Systems; 1:300). Double immunofluorescent staining was performed using Bond RX (Leica Biosystems) according to manufacturer protocols with the following primary antibodies: antibody against astrovirus capsid protein [13,17] (rabbit polyclonal; 1:500); anti-MAP2 (mouse monoclonal; Synaptic Systems; 1:300), and anti-synaptophysin (mouse monoclonal [SY38]; Abcam; 1:10). Host appropriate secondary antibodies were labeled with a red fluorescent dye Alexa Flour 594 (Life Technologies; 1:300) or biotinylated secondary antibody (Vector Laboratories; 1:200) and green fluorochrome streptavidin 488 (Life Technologies; 1:500). Detection was completed using the Bond Research Detection kit (Leica Biosystems; CAT# DS9455). Nuclei were counterstained with DAPI (Vector Laboratories), and sections were mounted with ProLong Gold anti-fade reagent (Invitrogen). EverBrite TrueBlack mounting medium (Biotium) was used to attempt to quench the lipofuscin autofluorescence according to manufacturer protocol with a limited success.

### Image acquisition and analysis

Brightfield colorimetric immunohistochemistry and H&E-stained sections were scanned at x40 magnification using the ScanScope AT2 (Leica Biosystems), and ImageScope software was used for digital slide image analysis. Neuronal virus burden was quantified using brightfield colorimetric immunohistochemistry for AstV capsid protein in each brain region of interest (2–6 sections per each) based on the counts of the AstV-positive (AstV+) neurons and their projections. The counts for AstV+ Purkinje cell bodies/dendrites in the cerebellar cortex were normalized to the length (mm) of the Purkinje cell layer (6 folia). The counts for AstV+ neurons in all other brain regions were normalized to the tissue area (mm2) with sampling focused on the gray matter. Mantra Imaging System and InForm software (Akoya Biosciences) were used for multispectral immunofluorescent image acquisition, spectral unmixing, and generation of simulated H&E images based on tissue autofluorescence according to manufacturer protocols. Simulated H&E images based on tissue autofluorescence were used as a tissue topographical reference for immunofluorescent images and to aid in identification of autofluorescent lipofuscin granules in corresponding immunofluorescent images.

## Supporting information

S1 Table Reported premorbid conditions and neuropathology associated with AstV-ND in humans and animals. (DOCX)

S2 Table Identification of astrovirus sequences mapped to a reference VA1/HMO clade astrovirus among the reads in brain samples based on RNA-seq data. (DOCX)

S1 Fig Detection of astrovirus in the brain of an immunocompromised patient with an encephalitis. (**A**) Fold change relative to control for probes to virus families hybridizing to RNA from the brain of the patient with AstV-NIH. (**B**) Detection of the astrovirus RNA in the brain of the patient with AstV-NIH by PCR. (**C** and **D**) In situ hybridization signals (magenta-red) in indicated brain tissue samples using the negative strand astrovirus RNA probe (upper panels) or RNA probes for actin as controls (lower panels).
(PDF)

S2 Fig Maximum-likelihood phylogeny of the mammal and avian astroviruses. The circular dendrogram is constructed from the ORF2/capsid precursor protein sequences of 229 astroviruses retrieved from the NCBI protein sequence database (see Materials and Methods for details). Thick branches were significantly supported (bootstrap percentage > 0.70) by the data. Mammalian and avian lineages are labeled at their most basal branches. The color labeling (indicated in the top right corner) is assigned based on the type of samples in which the virus was detected. Three astroviruses belonging to the Human VA1 clade that are associated with this study are indicated by bold red color. Other clades of human astroviruses are indicated in bold black color on the periphery (right side) of the dendrogram. Human astroviruses associated with CNS infections are indicated by black dots.
(PDF)

S3 Fig Clustering analysis of AstV-ND and normal control brain samples. The heatmap of the sample-to-sample distances in the transformed reads count matrix (dseq2). Samples that clustered together are highlighted by the same colors on the right and within the matrix. The pairs of AstV-ND and normal control samples chosen to identify DEGs for this study are shown on the right by arrows connecting relevant samples.
(PDF)

S1 File gProfiler Multiquery Downregulated Genes. (XLSX)

S2 File gProfiler Multiquery Upregulated Genes. (XLSX)
