# Supplementary material for: Pathogenesis and outcome of VA1 astrovirus infection in the human brain are defined by disruption of neural functions and imbalanced host immune responses
Source: PLoS Pathog. 2023 Aug 18;19(8):e1011544. doi: 10.1371/journal.ppat.1011544 (PMC10438012; doi:10.1371/journal.ppat.1011544)
Supplement: S1 Table — (DOCX) [file ppat.1011544.s001.docx]

**S1 Table.**

Reported premorbid conditions and neuropathology associated with AstV-ND in humans and animals.

| **Underlying disease** | **Infected CNS regions/CSF** | **Infected**  **cells** | **Diagnosis and neuropathological findings^a^** | **Outcome of AstV-ND (n)^b^** | **Reference** |
| --- | --- | --- | --- | --- | --- |
| **Humans** |  |  |  |  |  |
| X-linked agamma- globulinemia | Brainstem, cerebellum,  frontal cortex,  basal ganglia | Astrocytes  Neurons | Encephalitis: Astrocytosis (GFAP); microgliosis (CD68); T-cell perivascular/parenchymal infiltration (CD3); neuronal loss; axonal swelling; myelin degeneration | Fatal (1) | Quan et al, 2010 [[1](#_ENREF_1)]^c^ |
| Severe primary combined immunodeficiency | Brainstem, cerebellum,  basal ganglia, hippocampus | NR | Meningoencephalitis: Leptomeningeal/ periventricular inflammatory cell infiltration; necrosis; inflammatory cells not phenotyped | Fatal (1) | Wunderli et al, 2011 [[2](#_ENREF_2)] |
| Chronic lymphocytic leukemia | Frontal cortex | Neurons | Encephalitis: Astrocytosis (GFAP); microgliosis (CD68); T-cell parenchymal infiltration (CD3) | Fatal (1) | Naccache et al, 2015 [[3](#_ENREF_3)]^c^ |
| X-linked agamma-globulinemia | Frontal cortex | NR | Encephalitis: Microgliosis (IBA1); T-cell peri-vascular /parenchymal infiltration (CD3/CD8) | Recovery (1) | Fremond et al, 2015 [[4](#_ENREF_4)]^c^ |
| Cartilage hair hypoplasia | Cerebral cortex | Neurons | Encephalitis: Astrocytosis (GFAP); microgliosis (CD68); T-cell minimal infiltration (CD3);  neuronal apoptosis | Fatal (1) | Brown et al, 2015 [[5](#_ENREF_5)]^c^ |
| Acute myeloid leukemia | Unspecified | NR | Encephalitis: Hypercellularity; perivascular/ leptomenin-geal inflammatory cell infiltration; inflammatory cells not phenotyped | Fatal (1) | Lum et al, 2016 [[6](#_ENREF_6)]^c^ |
| Acute myeloid leukemia | Hypothalamic region | NR | Encephalitis: Myelin breakdown and neuronal microvesicular inclusions | Recovery (1) | Krol al, 2021 [[7](#_ENREF_7)] ^c^ |
| Acute myeloid leukemia | CSF | NR | Meningitis: NR | Fatal (1) | Cordey et al, 2016 [[8](#_ENREF_8)] |
| Congenital aplastic anemia | CSF | NR | Encephalopathy: NR | Recovery (1) | Sato et al, 2016 [[9](#_ENREF_9)] |
| None | CSF | NR | Meningitis NR | Recovery (1) | Cordey et al, 2016 [[8](#_ENREF_8)] |
| None | CSF | NR | Encephalitis: NR | Recovery (1) | Koukou et al, 2019 [[10](#_ENREF_10)] |
| None | CSF | NR | Meningitis: NR | Unknown (1) | Wilson et al, 2019 [[11](#_ENREF_11)] |
| B-cell acute lympho- blastic leukemia | CSF | NR | Encephalitis: NR | Recovery (1) | Bami et al, 2022 [[12](#_ENREF_12)] ^c^ |
| Acute myeloid leukemia | CSF | NR | Encephalitis: NR | Fatal (1) | Bami et al, 2022 [[12](#_ENREF_12)] ^c^ |
| **Cattle** |  |  |  |  |  |
| Unknown | Cerebellum, brainstem,  spinal cord, thalamus,  basal ganglia, cerebral cortex | Neurons | Neuronal degeneration; microgliosis; astrocytosis; perivascular infiltration (lymphocytes, macrophages, and plasma cells); inflammatory cells not phenotyped | Euthanasia | Li et al, 2013 [[13](#_ENREF_13)] |
| Unknown | Brainstem, cerebellum,  cerebrum | Neurons | Degeneration of neurons; microgliosis; perivascular infiltration; inflammatory cells not phenotyped | Euthanasia | Selimovic-Hamza et al, 2016, 1017 [[14](#_ENREF_14), [15](#_ENREF_15)] |
| Unknown | Medulla | Neurons | Degeneration of neurons; microgliosis;  inflammatory cells not phenotyped | Euthanasia | Bouzalas at, 2014 [[16](#_ENREF_16)] |
| **Pigs** |  |  |  |  |  |
| Unknown | Spinal cord, brainstem,  cerebellum, cerebral cortex | Neurons | Degeneration of neurons; microgliosis; perivascular/leptomeningeal infiltration;  inflammatory cells not phenotyped | Euthanasia | Boros et al. 2017 [[17](#_ENREF_17)] |
| **Sheep** |  |  |  |  |  |
| Unknown | Cerebellum, spinal cord, cerebrum | NR | NR | Euthanasia | Pfaff et al. 2016 [[18](#_ENREF_18)] |
| **Muskox** |  |  |  |  |  |
| Unknown | Spinal cord, midbrain | Unspecified | Perivascular cuffs; neuronal degeneration; gliosis; inflammatory cells not phenotyped | Euthanasia | Boujon et al, 2019 [[19](#_ENREF_19)] |
| **Alpaca** |  |  |  |  |  |
| Unknown | Spinal cord, brainstem,  cerebellum, thalamus,  cerebral cortex, hippocampus | Neurons | Predominantly lymphocytic perivascular cuffs; neuronal degeneration; gliosis; axonal degeneration, inflammatory cells not phenotyped | Euthanasia | Küchler et al, 2021 [[20](#_ENREF_20)] |
| **Mink** |  |  |  |  |  |
| Unknown | Unspecified | NR | NR | Euthanasia | Blomstrom et al, 2010 [[21](#_ENREF_21)] |

^a^ Neuropathological findings in humans are reported from postmortem or biopsy brain tissue samples in fatal or recovery cases, respectively. ^b^ Number of reported AstV-ND cases in humans. ^c^ Genotype of the astrovirus causing infection of the human CNS: Human VA1 clade. NR, not reported.

**References for S1 Table:**

1. Quan PL, Wagner TA, Briese T, Torgerson TR, Hornig M, Tashmukhamedova A, et al. Astrovirus encephalitis in boy with X-linked agammaglobulinemia. Emerging infectious diseases. 2010;16(6):918-25. Epub 2010/05/29. doi: 10.3201/eid1606.091536. PubMed PMID: 20507741; PubMed Central PMCID: PMCPMC4102142.

2. Wunderli W, Meerbach A, Güngör T, Berger C, Greiner O, Caduff R, et al. Astrovirus infection in hospitalized infants with severe combined immunodeficiency after allogeneic hematopoietic stem cell transplantation. PloS one. 2011;6(11):e27483. Epub 2011/11/19. doi: 10.1371/journal.pone.0027483. PubMed PMID: 22096580; PubMed Central PMCID: PMCPMC3214048.

3. Naccache SN, Peggs KS, Mattes FM, Phadke R, Garson JA, Grant P, et al. Diagnosis of neuroinvasive astrovirus infection in an immunocompromised adult with encephalitis by unbiased next-generation sequencing. Clinical infectious diseases : an official publication of the Infectious Diseases Society of America. 2015;60(6):919-23. Epub 2015/01/13. doi: 10.1093/cid/ciu912. PubMed PMID: 25572898; PubMed Central PMCID: PMCPMC4345816.

4. Frémond ML, Pérot P, Muth E, Cros G, Dumarest M, Mahlaoui N, et al. Next-Generation Sequencing for Diagnosis and Tailored Therapy: A Case Report of Astrovirus-Associated Progressive Encephalitis. Journal of the Pediatric Infectious Diseases Society. 2015;4(3):e53-7. Epub 2015/09/26. doi: 10.1093/jpids/piv040. PubMed PMID: 26407445.

5. Brown JR, Morfopoulou S, Hubb J, Emmett WA, Ip W, Shah D, et al. Astrovirus VA1/HMO-C: an increasingly recognized neurotropic pathogen in immunocompromised patients. Clinical infectious diseases : an official publication of the Infectious Diseases Society of America. 2015;60(6):881-8. Epub 2015/01/13. doi: 10.1093/cid/ciu940. PubMed PMID: 25572899; PubMed Central PMCID: PMCPMC4345817.

6. Lum SH, Turner A, Guiver M, Bonney D, Martland T, Davies E, et al. An emerging opportunistic infection: fatal astrovirus (VA1/HMO-C) encephalitis in a pediatric stem cell transplant recipient. Transplant infectious disease : an official journal of the Transplantation Society. 2016;18(6):960-4. Epub 2016/10/28. doi: 10.1111/tid.12607. PubMed PMID: 27632248.

7. Król L, Turkiewicz D, Nordborg K, Englund E, Stenberg L, Karlsson Lindsjö O, et al. Astrovirus VA1/HMO encephalitis after allogeneic hematopoietic cell transplantation: Significant role of immune competence in virus control. Pediatric blood & cancer. 2021;68(12):e29286. Epub 2021/08/20. doi: 10.1002/pbc.29286. PubMed PMID: 34411414.

8. Cordey S, Vu DL, Schibler M, L'Huillier AG, Brito F, Docquier M, et al. Astrovirus MLB2, a New Gastroenteric Virus Associated with Meningitis and Disseminated Infection. Emerging infectious diseases. 2016;22(5):846-53. Epub 2016/04/19. doi: 10.3201/eid2205.151807. PubMed PMID: 27088842; PubMed Central PMCID: PMCPMC4861523.

9. Sato M, Kuroda M, Kasai M, Matsui H, Fukuyama T, Katano H, et al. Acute encephalopathy in an immunocompromised boy with astrovirus-MLB1 infection detected by next generation sequencing. Journal of clinical virology : the official publication of the Pan American Society for Clinical Virology. 2016;78:66-70. Epub 2016/03/19. doi: 10.1016/j.jcv.2016.03.010. PubMed PMID: 26991054.

10. Koukou G, Niendorf S, Hornei B, Schlump JU, Jenke AC, Jacobsen S. Human astrovirus infection associated with encephalitis in an immunocompetent child: a case report. Journal of medical case reports. 2019;13(1):341. Epub 2019/11/24. doi: 10.1186/s13256-019-2302-6. PubMed PMID: 31757225; PubMed Central PMCID: PMCPMC6874811.

11. Wilson MR, Sample HA, Zorn KC, Arevalo S, Yu G, Neuhaus J, et al. Clinical Metagenomic Sequencing for Diagnosis of Meningitis and Encephalitis. The New England journal of medicine. 2019;380(24):2327-40. Epub 2019/06/13. doi: 10.1056/NEJMoa1803396. PubMed PMID: 31189036; PubMed Central PMCID: PMCPMC6764751.

12. Bami S, Hidinger J, Madni A, Hargest V, Schultz-Cherry S, Cortez V, et al. Human Astrovirus VA1 Encephalitis in Pediatric Patients With Cancer: Report of 2 Cases and Review of the Literature. Journal of the Pediatric Infectious Diseases Society. 2022;11(9):408-12. Epub 2022/07/19. doi: 10.1093/jpids/piac045. PubMed PMID: 35849135.

13. Li L, Diab S, McGraw S, Barr B, Traslavina R, Higgins R, et al. Divergent astrovirus associated with neurologic disease in cattle. Emerging infectious diseases. 2013;19(9):1385-92. Epub 2013/08/24. doi: 10.3201/eid1909.130682. PubMed PMID: 23965613; PubMed Central PMCID: PMCPMC3810933.

14. Selimovic-Hamza S, Bouzalas IG, Vandevelde M, Oevermann A, Seuberlich T. Detection of Astrovirus in Historical Cases of European Sporadic Bovine Encephalitis, Switzerland 1958-1976. Frontiers in veterinary science. 2016;3:91. Epub 2016/10/27. doi: 10.3389/fvets.2016.00091. PubMed PMID: 27781208; PubMed Central PMCID: PMCPMC5058262.

15. Selimovic-Hamza S, Boujon CL, Hilbe M, Oevermann A, Seuberlich T. Frequency and Pathological Phenotype of Bovine Astrovirus CH13/NeuroS1 Infection in Neurologically-Diseased Cattle: Towards Assessment of Causality. Viruses. 2017;9(1). Epub 2017/01/21. doi: 10.3390/v9010012. PubMed PMID: 28106800; PubMed Central PMCID: PMCPMC5294981.

16. Bouzalas IG, Wüthrich D, Walland J, Drögemüller C, Zurbriggen A, Vandevelde M, et al. Neurotropic astrovirus in cattle with nonsuppurative encephalitis in Europe. Journal of clinical microbiology. 2014;52(9):3318-24. Epub 2014/07/06. doi: 10.1128/jcm.01195-14. PubMed PMID: 24989603; PubMed Central PMCID: PMCPMC4313157.

17. Boros Á, Albert M, Pankovics P, Bíró H, Pesavento PA, Phan TG, et al. Outbreaks of Neuroinvasive Astrovirus Associated with Encephalomyelitis, Weakness, and Paralysis among Weaned Pigs, Hungary. Emerging infectious diseases. 2017;23(12):1982-93. Epub 2017/11/18. doi: 10.3201/eid2312.170804. PubMed PMID: 29148391; PubMed Central PMCID: PMCPMC5708238.

18. Pfaff F, Schlottau K, Scholes S, Courtenay A, Hoffmann B, Höper D, et al. A novel astrovirus associated with encephalitis and ganglionitis in domestic sheep. Transboundary and emerging diseases. 2017;64(3):677-82. Epub 2017/02/23. doi: 10.1111/tbed.12623. PubMed PMID: 28224712.

19. Boujon CL, Koch MC, Kauer RV, Keller-Gautschi E, Hierweger MM, Hoby S, et al. Novel encephalomyelitis-associated astrovirus in a muskox (Ovibos moschatus): a surprise from the archives. Acta veterinaria Scandinavica. 2019;61(1):31. Epub 2019/06/27. doi: 10.1186/s13028-019-0466-0. PubMed PMID: 31234899; PubMed Central PMCID: PMCPMC6591865.

20. Küchler L, Rüfli I, Koch MC, Hierweger MM, Kauer RV, Boujon CL, et al. Astrovirus-Associated Polioencephalomyelitis in an Alpaca. Viruses. 2020;13(1). Epub 2021/01/06. doi: 10.3390/v13010050. PubMed PMID: 33396858; PubMed Central PMCID: PMCPMC7824642.

21. Blomström AL, Widén F, Hammer AS, Belák S, Berg M. Detection of a novel astrovirus in brain tissue of mink suffering from shaking mink syndrome by use of viral metagenomics. Journal of clinical microbiology. 2010;48(12):4392-6. Epub 2010/10/12. doi: 10.1128/jcm.01040-10. PubMed PMID: 20926705; PubMed Central PMCID: PMCPMC3008476.
