# Supplementary material for: Pathogenesis and outcome of VA1 astrovirus infection in the human brain are defined by disruption of neural functions and imbalanced host immune responses
Source: PLoS Pathog. 2023 Aug 18;19(8):e1011544. doi: 10.1371/journal.ppat.1011544 (PMC10438012; doi:10.1371/journal.ppat.1011544)
Supplement: S2 Table — (DOCX) [file ppat.1011544.s002.docx]

**S2 Table.**

Identification of astrovirus sequences mapped to a reference VA1/HMO clade astrovirus among the reads in brain samples based on RNA-seq data.

| **Brain tissue sample** | **Start**  **position** | **End**  **position**  **(sequence length)** | **Number of reads** | **Number of covered**  **bases** | **Percent of covered bases** | **Mean depth of coverage** | **Mean base quality in covered region** | **Mean map quality of selected reads** |
| --- | --- | --- | --- | --- | --- | --- | --- | --- |
| AstV-ND-1-NIH (unknown site) | 1 | 6586 | 4670 | 6546 | 99.4 | 53.8 | 35.3 | 32.2 |
| AstV-ND-2-NY (brainstem) | 1 | 6586 | 38 | 452 | 6.9 | 0.4 | 35.4 | 31.5 |
| AstV-ND-3-France (frontal cortex) | 1 | 6586 | 202 | 3627 | 55.1 | 2.3 | 35.3 | 21.8 |
| Normal control (thalamus) | 1 | 6586 | 0 | 0 | 0 | 0 | 0 | 0 |
| Normal control (brainstem) | 1 | 6586 | 0 | 0 | 0 | 0 | 0 | 0 |
| Normal control (brainstem) | 1 | 6586 | 0 | 0 | 0 | 0 | 0 | 0 |
| Normal control (frontal cortex) | 1 | 6586 | 0 | 0 | 0 | 0 | 0 | 0 |
| Normal control (frontal cortex) | 1 | 6586 | 0 | 0 | 0 | 0 | 0 | 0 |
