# Supplementary figures and images for: Pathogenesis and outcome of VA1 astrovirus infection in the human brain are defined by disruption of neural functions and imbalanced host immune responses
Source: PLoS Pathog. 2023 Aug 18;19(8):e1011544. doi: 10.1371/journal.ppat.1011544 (PMC10438012; doi:10.1371/journal.ppat.1011544)

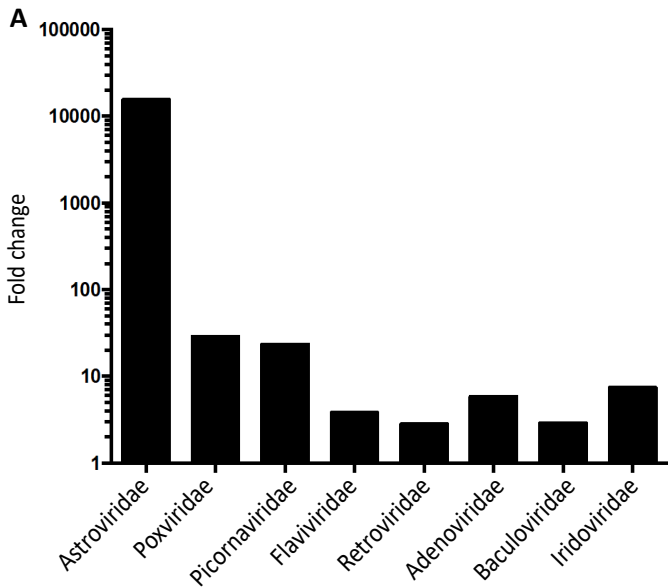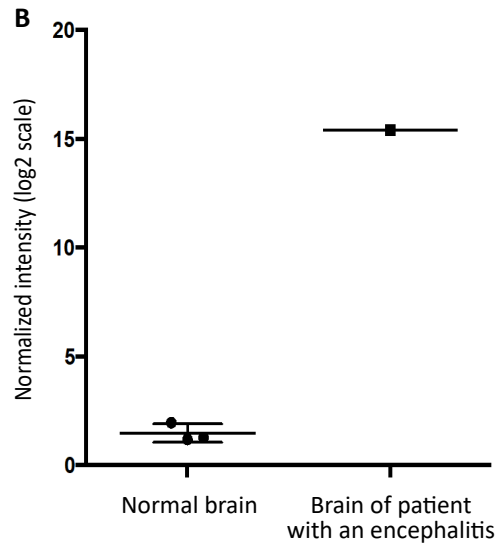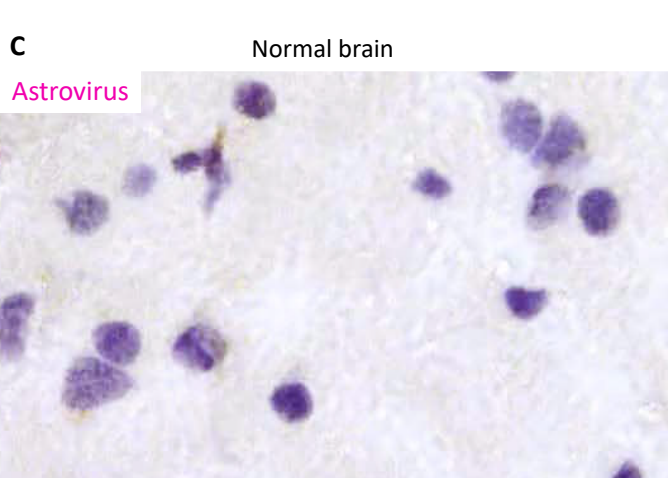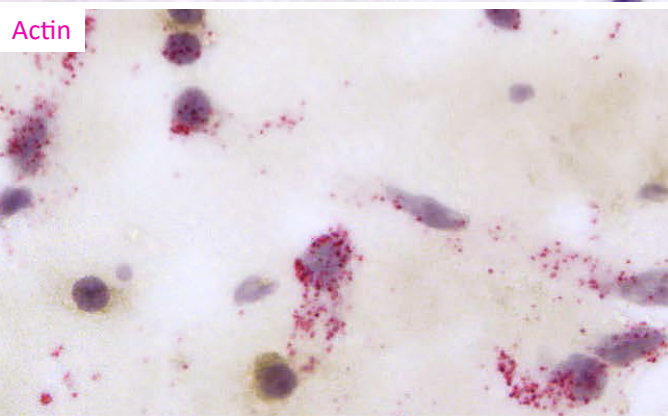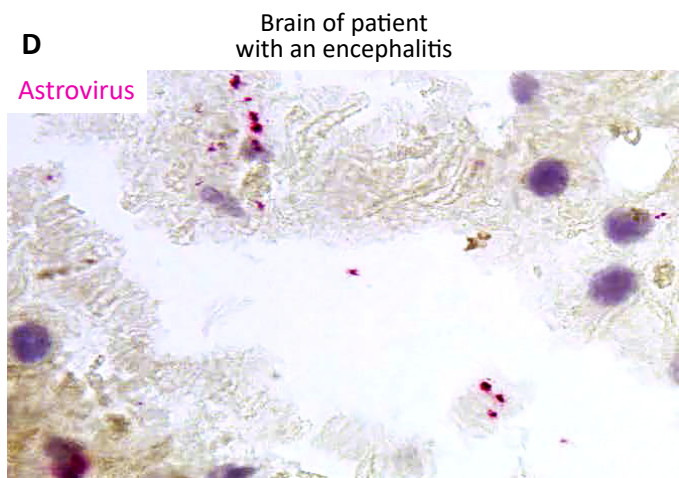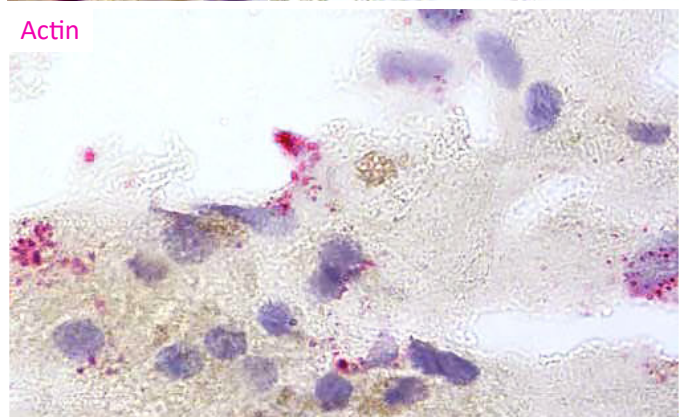

Supplement: S1 Fig — (A) Fold change relative to control for probes to virus families hybridizing to RNA from the brain of the patient with AstV-NIH. (B) Detection of the astrovirus RNA in the brain of the patient with AstV-NIH by PCR. (C and D) In situ hybridization signals (magenta-red) in indicated brain tissue samples using the negative strand astrovirus RNA probe (upper panels) or RNA probes for actin as controls (lower panels). (PDF) [file ppat.1011544.s003.pdf]

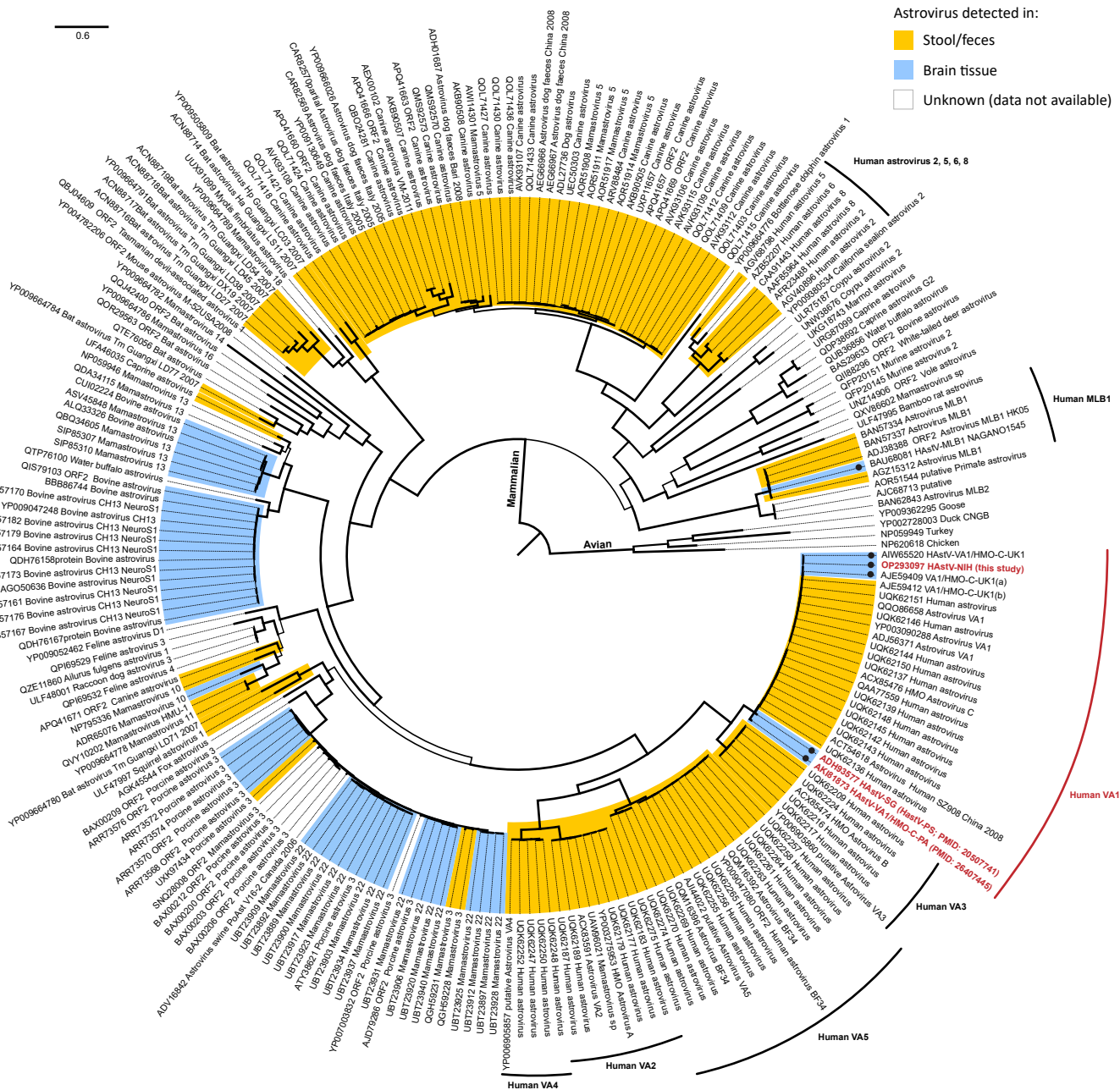

Supplement: S2 Fig — The circular dendrogram is constructed from the ORF2/capsid precursor protein sequences of 229 astroviruses retrieved from the NCBI protein sequence database (see Materials and Methods for details). Thick branches were significantly supported (bootstrap percentage > 0.70) by the data. Mammalian and avian lineages are labeled at their most basal branches. The color labeling (indicated in the top right corner) is assigned based on the type of samples in which the virus was detected. Three astroviruses belonging to the Human VA1 clade that are associated with this study are indicated by bold red color. Other clades of human astroviruses are indicated in bold black color on the periphery (right side) of the dendrogram. Human astroviruses associated with CNS infections are indicated by black dots. (PDF) [file ppat.1011544.s004.pdf]

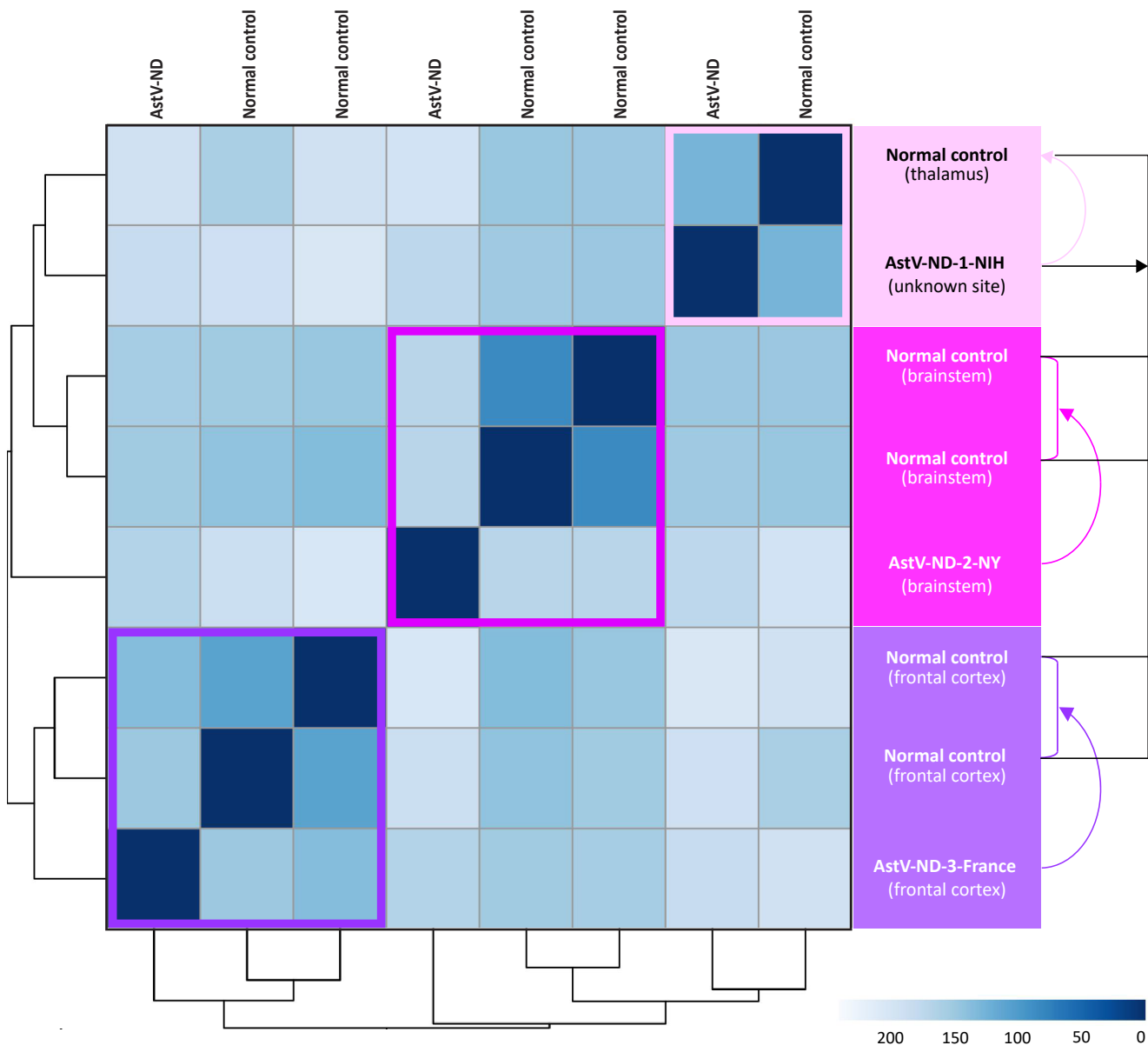

Supplement: S3 Fig — The heatmap of the sample-to-sample distances in the transformed reads count matrix (dseq2). Samples that clustered together are highlighted by the same colors on the right and within the matrix. The pairs of AstV-ND and normal control samples chosen to identify DEGs for this study are shown on the right by arrows connecting relevant samples. (PDF) [file ppat.1011544.s005.pdf]
